# Neuroinflammation, Microglia and Implications for Retinal Ganglion Cell Survival and Axon Regeneration in Traumatic Optic Neuropathy

**DOI:** 10.3389/fimmu.2022.860070

**Published:** 2022-03-04

**Authors:** Ngan Pan Bennett Au, Chi Him Eddie Ma

**Affiliations:** ^1^ Department of Neuroscience, City University of Hong Kong, Hong Kong, Hong Kong SAR, China; ^2^ City University of Hong Kong Shenzhen Research Institute, Shenzhen, China

**Keywords:** optic neuropathy, microglia, retinal degeneration, astrocyte, Müller cell, neuroinflammation

## Abstract

Traumatic optic neuropathy (TON) refers to a pathological condition caused by a direct or indirect insult to the optic nerves, which often leads to a partial or permanent vision deficit due to the massive loss of retinal ganglion cells (RGCs) and their axonal fibers. Retinal microglia are immune-competent cells residing in the retina. In rodent models of optic nerve crush (ONC) injury, resident retinal microglia gradually become activated, form end-to-end alignments in the vicinity of degenerating RGC axons, and actively internalized them. Some activated microglia adopt an amoeboid morphology that engulf dying RGCs after ONC. In the injured optic nerve, the activated microglia contribute to the myelin debris clearance at the lesion site. However, phagocytic capacity of resident retinal microglia is extremely poor and therefore the clearance of cellular and myelin debris is largely ineffective. The presence of growth-inhibitory myelin debris and glial scar formed by reactive astrocytes inhibit the regeneration of RGC axons, which accounts for the poor visual function recovery in patients with TON. In this Review, we summarize the current understanding of resident retinal microglia in RGC survival and axon regeneration after ONC. Resident retinal microglia play a key role in facilitating Wallerian degeneration and the subsequent axon regeneration after ONC. However, they are also responsible for producing pro-inflammatory cytokines, chemokines, and reactive oxygen species that possess neurotoxic effects on RGCs. Intraocular inflammation triggers a massive influx of blood-borne myeloid cells which produce oncomodulin to promote RGC survival and axon regeneration. However, intraocular inflammation induces chronic neuroinflammation which exacerbates secondary tissue damages and limits visual function recovery after ONC. Activated retinal microglia is required for the proliferation of oligodendrocyte precursor cells (OPCs); however, sustained activation of retinal microglia suppress the differentiation of OPCs into mature oligodendrocytes for remyelination after injury. Collectively, controlled activation of retinal microglia and infiltrating myeloid cells facilitate axon regeneration and nerve repair. Recent advance in single-cell RNA-sequencing and identification of microglia-specific markers could improve our understanding on microglial biology and to facilitate the development of novel therapeutic strategies aiming to switch resident retinal microglia’s phenotype to foster neuroprotection.

## Introduction

The retina is a highly organized tissue that functions to convey visual signals to the brain for both image-forming and non-image-forming visual function. The mammalian retina has more than 60 functionally distinct cell types that mainly reside in three different cellular layers (1, 2). The outer nuclear layer (ONL) consists of two types of photoreceptors: the rods and the cones. Human retina consists of approximately 92 million rod and 4.6 million cone photoreceptors (3), which are responsible for the detection of light stimuli and pass visual information to interneurons. The inner nuclear layer (INL) consists of three major types of interneurons (bipolar cells, horizontal cells and amacrine cells) (4), which process visual information and pass it to retinal ganglion cells (RGCs). The ganglion cell layer (GCL) consists of mainly RGCs which accounts for approximately 1% of the total cells (i.e. ~1.2 million) in the human retina (5). RGCs are primed to deliver visual information to the brain through the optic nerves (6, 7). Recent single-cell RNA-sequencing revealed more than 46 distinct subtypes of RGCs in the adult retina (8, 9). As an extension to the brain and a part of the CNS, mammalian retina also consists of three main types of glial cells: Müller cells, astrocytes and resident microglia; all of these glial cells are essential to maintain retinal homeostasis (10). Müller cells are highly specialized macroglial cells that span across the entire retinae and represent the largest population of glial cells in the mammalian retina (~80-90% of total glial cells) (11). Retinal astrocytes are another macroglial cells that are mainly found in the nerve fiber layer (NFL) in the mammalian retina (12). Retinal microglia were first identified by Lopez Enrique (13) and Marchesani (14) in 1926, and are mainly found in the NFL and IPL of retinae as well as in the optic tract accounting for ~5-20% of total glial cells in the retina (15–17). Retinal microglia possess distinct morphological and immunohistochemical properties, which distinguish them from horizontal cells and macroglial cells in the retina (18). In most cases, microglial cell markers such as CD11b (also known as OX42), F4/80, CD68 (antibody clone ED-1 for detection of rat CD68), and ionized calcium-binding adapter molecule 1 (IBA1) (19, 20) can be used to differentiate microglia from other cells. Retinal microglia are described as active surveillance and immunocompetent cells residing in various layers of healthy retinae including NFL, GCL, inner plexiform layer (IPL), outer plexiform layer (OPL), and INL (10).

**Figure 1:**
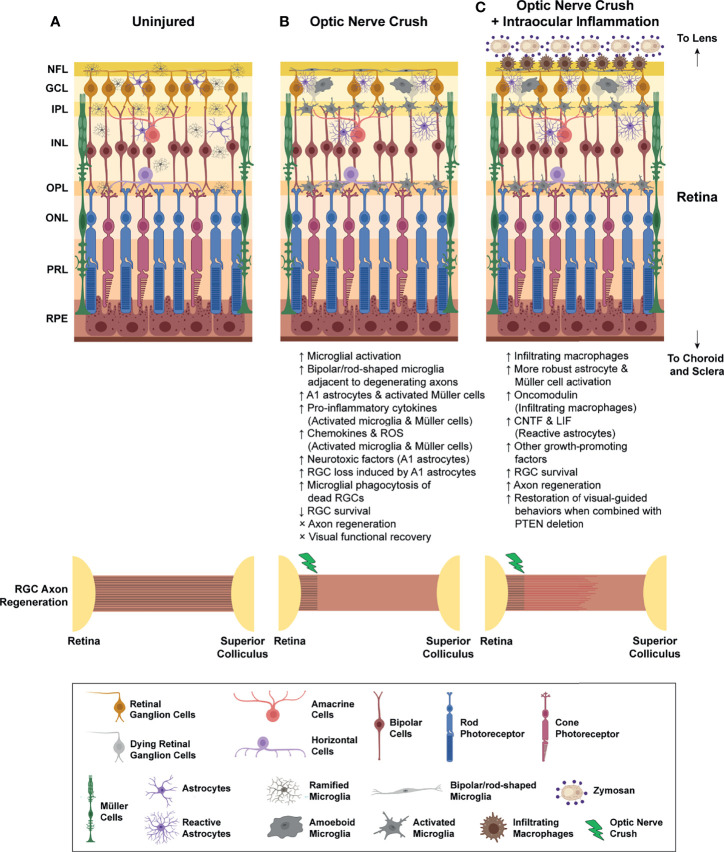
Inflammatory responses after optic nerve crush (ONC) injury. **(A)** In the intact retina, retinal microglia usually resided in NFL, GCL, IPL, INL, and OPL. These resident retinal microglia adopt ramified morphology with which they constantly send and withdraw their processes to sense the surrounding microenvironment. Retinal astrocytes and Müller cells are the two major macroglial cells that resided in retinae for the maintenance of retinal homeostasis. **(B)** Optic nerve crush (ONC) triggers the activation of resident retinal microglia, as well as retinal astrocytes and Müller cells. Some of the microglia transform into bipolar/rod-shaped microglia and colonize adjacent to the degenerating RGC axons in NFL. These bipolar/rod-shaped microglia actively internalized these degenerating axons after ONC. Some activated microglia adopt amoeboid morphology and actively engulf the dead RGCs in GCL. Also, these activated retinal microglia produce a broad spectrum of pro-inflammatory cytokines (e.g. IL-1β, IL-6 and TNF-α), chemokines (CCL2, CCL3 and CCL5), and reactive oxygen species (ROS), which induced reactive astrocytes to adopt a neurotoxic A1 phenotype and increased production of neurotoxic factors. Activated Müller cells are also responsible for the production of pro-inflammatory cytokines in the injured retinae. Collectively, the number of survived RGCs gradually declined over time, and most of these survived RGCs failed to regenerate across the lesion site, leading to a permanent vision loss after ONC. **(C)** Intraocular inflammation induced by zymosan or curdlan treatment, or lens injury triggers a massive influx of blood-borne myeloid cells, and robust astrocyte and Müller cell activation. The infiltrating myeloid cells produce a large quantity of growth-promoting factors, such as oncomodulin, which promoted RGC survival after ONC. The reactive astrocytes also elevate the production of CNTF and LIF after intraocular inflammation. More importantly, the macrophage- and astrocyte-derived growth-promoting factors stimulate robust axon regeneration which enabled a partial restoration of some visual-guided behaviors when combined with other manipulations, such as PTEN deletion. NFL, nerve fibre layer; GCL, ganglion cell layer; IPL, inner plexiform layer; INL, inner nuclear layer; OPL, outer plexiform layer; ONL, outer nuclear layer; PRL, photoreceptor layer; RPE, retinal pigment epithelium; RGC, retinal ganglion cell; ROS, reactive oxygen species. CNTF, ciliary neurotrophic factor; LIF, leukemia inhibitory factor.

The dynamic nature of microglia and their vast phenotypic diversity as reflected by a variety of microglial morphologies in different brain regions, and under diverse pathological conditions. In rodent retinae, microglia started to appear in the GCL and neuroblastic layer of developing retinae at embryonic day 11.5 (E11.5) (21). These resident retinal microglia adopt an amoeboid-like phenotype and actively phagocytized dying RGCs until postnatal day 5 (P5) (21, 22), a time when programed cell death of RGCs has reached its peak during the development of retinae (23). After that, amoeboid microglia transform into a ‘surveying’ phenotype with compact cell bodies and long branching processes, known as ‘ramified microglia’. Ramified microglia mainly reside in the GCL, IPL, INL, and OPL of the healthy adult retina (24) (**Figure 1A**). Ramified microglia are active and highly motile surveillant cells with their processes undergoing constant extension and retraction to sense the surrounding microenvironment for perturbations (25). Once subtle damage to the tissue is detected, ramified microglia quickly thicken their processes, transform into amoeboid microglia with enlarged cell bodies, and colonize in close proximity to lesions shortly after injuries (26). Amoeboid microglia are regarded as ‘activated microglia’ that are responsible for the production of pro-inflammatory (M1) and anti-inflammatory (M2) cytokines, chemokines, and various neurotrophic factors. They are also involved in the elimination of dysregulated synapses, phagocytosis of dead cells and cellular debris, and antigen presentation (17, 27). Acute and controlled activation of microglia favor neuroprotection, limit secondary tissue damages and facilitate nerve repair. In contrast, persistent microglial activation often leads to excessive production of neurotoxic pro-inflammatory (M1) cytokines and other inflammatory mediators that exacerbate secondary tissue damages. Activation of resident retinal microglia is a common hallmark of many retinal degenerative disorders, including traumatic optic neuropathy, age-related macular degeneration, glaucoma, retinitis pigmentosa, uveoretinitis and diabetic retinopathies (15, 28–31). Targeting microglial activation might therefore represent an attractive therapeutic target for the development of novel therapies in CNS injuries.

In this Review, we summarize the current understanding of resident retinal microglia after traumatic optic neuropathy (TON). Compelling evidence suggests that activation of resident retinal microglia and recruitment of infiltrating macrophages are essential for effective clearance of myelin debris and production of growth factors that facilitate nerve repair. In contrast, sustained and unresolved neuroinflammation contributed to production of neurotoxic pro-inflammatory cytokines and persistent secondary tissue damages. To this end, we aim to provide a novel insight into the development of new therapeutic strategies for TON.

## Neuroinflammation and Microglial Activation After Traumatic Optic Neuropathy

TON refers to any direct or indirect insult which causes damage to the optic nerves and its axonal fibres. Permanent visual impairments are seen most commonly in patients resulting from a massive loss in RGCs and axonal connections with their brain after TON (32). In addition, the remaining viable RGCs failed to regenerate axons spontaneously which further limited the restoration of visual function after injuries (33, 34). The availability of well-established rodent models of optic nerve crush (ONC) for TON and glaucomatous injury (35), which enable researchers to study molecular machinery for neuronal survival and axon regeneration for recovery of visual function. In the past decades, a broad spectrum of intrinsic regulators for pro-regenerative growth, and factors that involved in glial inhibition have been identified. Manipulation of these factors enabled sustained and long-distance axon regeneration after ONC (33, 36, 37). However, the roles of resident microglia in RGC survival and axon regeneration after ONC remained largely unexplored.

### Microglial Phagocytosis in Wallerian Degeneration and Axon Regeneration After Optic Nerve Injuries

After ONC, the resident microglia become activated, adopting a reactive phenotype characterized by enlarged cell bodies and shortened processes, and colonized progressively the GCL, IPL and OPL (38, 39) (**Figure 1B**) as well as the optic nerves (39). Blood-borne macrophages with large and round cell bodies are also recruited to the crushed site of the optic nerve after ONC (40). The resident retinal microglia and infiltrating macrophages are known to be involved in the degradation and removal of myelin debris from the injured optic nerve, a process known as Wallerian degeneration (41). In the peripheral nervous system (PNS), after degradation of axons, myelin sheaths are collapsed, degenerated, and cleared effectively starting within hours and completed in days after peripheral nerve injury (PNI) (42–45). Within hours after PNI, Schwann cells undergo rapid dedifferentiation and proliferation at the distal nerve stump (42). In parallel, a rapid influx of blood-borne macrophages towards the proximal and distal nerve stump occurs in the first 12 hours after PNI (40, 46). Infiltrating macrophages display a tremendous phagocytic capacity to remove myelin debris (43). Successful completion of Wallerian degeneration generates a growth-permissive environment, which enables regenerating motor axons to reinnervate motor endplates at the distal target muscles for motor function recovery after PNI (44, 47–49). In contrast to the PNS, Wallerian degeneration after CNS injuries occurs at an extremely slow rate. Oligodendrocytes are the glial cells responsible for clearance of myelin debris after CNS injuries. However, oligodendrocytes underwent apoptosis soon after ONC due to the loss of retinal inputs from RGCs (50, 51) and the oligodendrocyte precursor cells failed to proliferate as well (52). Injury to the optic nerve had no immediate effect on the immune cell populations, neither colonization of resident microglia, nor influx of infiltrating macrophages were observed in the distal nerve stump at 1 week post-ONC (53). Macrophages became more prominent throughout the optic nerve only at 8 weeks post-ONC (40, 41). In addition, activated retinal microglia displayed diminished phagocytic capacity to remove myelin debris from the degenerating optic nerve (54). As a result, clearance of myelin debris by the resident retinal microglia and infiltrating macrophages become largely ineffective which persists for months after ONC (40, 51).

Injury to the CNS is accompanied by reactive gliosis, inflammation and formation of glial scar. Scar tissue and associated deposition of extracellular matrix (ECM) molecules such as chondroitin sulfate proteoglycans (CSPG), and the presence of myelin-associated inhibitors (MAIs), among which Nogo-A, myelin-associated glycoprotein (MAG) and oligodendrocyte-myelin glycoprotein (OMgp) are the major players in CNS myelin that inhibit axon regeneration (36, 55). The reactive astrocytes and the formation of a glial scar (also known as astrocytic scar) facilitate the repair of damaged tissue by limiting the spread of inflammation and separating healthy tissue from pathology following injury. However, the persistence of scarring beyond the acute phase of injury can impede axon regeneration and long-term recovery (55–57). In response to ONC, reactive astrocytes can be found in the retinae (58, 59) and optic nerve (60, 61), producing growth-inhibitory molecules such as CSPG at the crushed site (60, 61). In the presence of glial scar, regenerating axons formed dystrophic and swollen retraction bulbs while trying to penetrate a glial scar, a phenomenon known as growth cone collapse (62). The formation of dystrophic growth cones stalls the axonal growth, and therefore virtually no axons could regenerate beyond the glial scar after ONC. Unfortunately, pharmaceutical blockade or genetic ablation of some of the known MAIs failed to facilitate sustained axon regeneration for function recovery after ONC (63, 64). More importantly, chronic glial scarring has been shown to transform resident microglia and infiltrating macrophages into a neurotoxic M1-like phenotype (65–67), further exacerbating axonal damage secondary to the myelin damage. Therefore, experimental therapies to increase macrophage infiltration by treating the rats with a group B-streptococcus exotoxin, exhibited a rapid infiltration of macrophages into the injured optic nerve and demonstrated a higher phagocytic capacity than the resident retinal microglia (54). The increase in macrophage influx around the crushed site led to the reduction of glial scar and reactive astrocytes, and the effective clearance of myelin debris facilitating axons to regenerate across the glial scar after ONC (68). Furthermore, pre-treating peripheral blood-borne macrophages with sciatic nerve segments activated macrophages and enhanced their phagocytic capacity. Subsequent transplantation of these activated macrophages into injured optic nerve facilitated myelin debris clearance within 7 days after optic nerve transection (69). This raise the possibility that by switching resident retinal microglia and infiltrating macrophage polarization to a neuroprotective M2-like phenotype with higher phagocytic capacity creates a growth permissive microenvironment for axon regeneration after ONC (70). In fact, M2 microglia produce anti-inflammatory cytokines and neurotrophic factors (71, 72) that are neuroprotective in nature promoting neuronal survival and repair after CNS injuries (73, 74). In another study, recombinant interferon-β (IFN-β) augmented the phagocytic capacity of primary microglia and contributed to the active removal of myelin debris by activated microglia *in vitro* (75). Further investigation is required to examine whether these factors would also prime resident retinal microglia for effective clearance of myelin debris and switch a hostile CNS microenvironment to a growth-permissive microenvironment after ONC.

The resident microglia express a range of surface receptors for recognition and phagocytosis of cell undergoing apoptosis (76). Accumulating evidence suggests that the clearance of myelin debris after CNS injuries involves the activation of complement signaling cascades (77–79). A recent study highlighted that complement activation in resident retinal microglia and infiltrating macrophages are pre-requisite for successful axon regeneration after ONC. Both complement proteins C1q and C3 as well as the complement receptor CR3, were markedly elevated in the optic nerve but not in the mouse retinae shortly after ONC (80). It has been reported that complement protein C1q and C3 promoted axon regeneration and function recovery after SCI (81, 82). Genetic ablation of C1q, C3 and CR3 receptors, completely abolished the growth-promoting effects induced by several manipulations known to promote axon regeneration in adult mice after ONC (80), including intraocular inflammation using zymosan treatment (83–86), zinc chelator TPEN to block the accumulation of mobile zinc ions in injured RGCs (87), and deletion of phosphatase and tensin homolog (PTEN) with oncomodulin treatment (88, 89). Resident microglia are known to express and produce both complement proteins C1q and C3 in the retina (90, 91). To address the roles of resident retinal microglia in the production of complement proteins and activation of complement signaling cascades, the authors attempted to deplete microglia using PLX5622 which allows the studies of RGC axon regeneration in the absence of microglia (80). PLX5622 and its structural homolog PLX3397 could eliminate virtually all microglia from the adult CNS through binding the colony-stimulating factor-1 receptor (CSF1R) (92–94). Both PLX5622 and PLX3397 becomes a valuable tool to study the roles of microglia in the pathogenesis of numerous neurodegenerative diseases such as Alzheimer’s Disease (93), Parkinson’s Disease (95), ischemic stroke (96), amyotrophic lateral sclerosis (97), and Charcot-Marie-Tooth disease (98). A recent study demonstrates that PLX5622 treatment eliminated most of the resident retinal microglia from injured optic nerve and retinae. Subsequently, the expressions of complement proteins C1q and C3, and CR3 receptor, were markedly reduced at the early time points after ONC suggesting that resident retinal microglia are the major source of these complement proteins. More importantly, activated resident retinal microglia with high expression of CR3 receptor, actively removed myelin debris at the lesion site after ONC to create a growth-permissive microenvironment for axon regeneration. In contrast, genetic ablation of CR3 receptor largely impaired the clearance of myelin debris at the lesion site and thus stalled axon regeneration (80). Interestingly, one study suggested that microglial depletion using PLX5622 did not affect lens injury-induced axon growth in adult mice after ONC (94). The reason for this discrepancy is unclear but could possibly be related to differences in the time points being analyzed (14 versus 21 days post-ONC). Nevertheless, another possible explanation is that lens injury induces a group of growth-promoting factors that are unavailable in the zymosan-induced intraocular inflammation, and the lens injury-specific growth-promoting factors support RGC axon regeneration even when the clearance of myelin debris was largely impaired in the absence of retinal microglia. Interestingly, a suboptimal dose of zymosan produced modest level of axon regeneration in C57BL/6 mice; however, administration of the same doses of zymosan in CAST/Ei mice sustained long-distance axons regenerated along the entire length of the optic nerve, reaching the optic chiasm (99). It suggests that genetically diverse inbred mouse strains with varied intrinsic growth capacity response very differently to lens injury and regenerate injured CNS axons at different extent. The reasons for this will require further investigation of the casual link between inflammation and retinal microglia responses across mouse strains.

### Detrimental Roles of Activated Retinal Microglia and Macroglia in RGC Loss and Axonal Degeneration After Optic Nerve Injuries

ONC triggers neuronal apoptosis in the RGCs (100), which activates resident retinal microglia and recruitment of infiltrating macrophages into the retinae and proximal nerve stump (59). In adult female rats, an increased number of microglia was detected in the retinae as early as 2 days after ONC and accompanied by increase production of neurotoxic pro-inflammatory cytokines in the retinae and proximal nerve stump, resulting in widespread neuronal loss and axon degeneration after ONC (38). The number of activated microglia gradually increased over time, with its peak at day 14 after ONC. Sustained microglial activation was observed in the retinae even 2 months after ONC and RGC survival was reduced to 7% (38). At the same time, the expression levels of M2 markers (CD206, Ym1 and Arg1) were substantially declined and expression levels of M1 markers (IL-1β, IL-6 and TNF-α) were elevated after prolonged activation of retinal microglia, which induced neuronal apoptosis and exacerbated secondary tissue damage (101, 102). It is well documented that inducible nitric oxide synthase (iNOS) expression level is upregulated in activated microglia, which can cause RGC death (103, 104). Profound microglial activation was also observed in the proximal nerve stump at day 3 post-ONC, with its peak at day 5 post-ONC, which was accompanied by elevated production of neurotoxic pro-inflammatory cytokine IL-1β and profound axonal damage (105).

ONC triggers not only the activation of resident retinal microglia, but also other macroglia in the retinae, including astrocytes and Müller cells. Growing evidence suggests that resident retinal microglia might interact with these macroglial cells to modulate RGC survival and axon regeneration after ONC (106). After ONC in rats, the intraocular glutamate level became significantly elevated in the first week of injury, which gradually returned to basal level at two weeks post-injury (107). The elevated intraocular glutamate induced activation of Müller cells which led to the release of adenosine triphosphate (ATP) (108), a well-known chemoattractant for microglia and an important mediator for injury-induced microglial responses (26). In primary culture of rat brain microglia, microglia were highly responsive to extracellular ATP with increased expression of neurotoxic pro-inflammatory cytokines IL-1β and TNF-α (109, 110). Co-culture of Müller cells and lipopolysaccharide (LPS)-activated retinal microglia, Müller cells became highly activated with prominent alternation in cell morphology and increased production of a number of neurotrophic factors and pro-inflammatory modulators, including glial cell-derived neurotrophic factor (GDNF), leukemia inhibitory factor (LIF), IL-1β, IL-6 and iNOS (111). Interestingly, conditioned medium derived from activated Müller cells further stimulated retinal microglia to produce more IL-1β, IL-6 and iNOS in cultures (111), which suggests a reciprocal signaling between Müller cells and resident retinal microglia to prolong the local inflammatory responses in the injured retinae. There is increasing evidence that resident retinal microglia might interact with retinal astrocytes directly after ONC. Indeed, a recent study showed that neurotoxic reactive astrocytes were primed by activated microglia in cultures. These effects were accomplished through increased secretion of pro-inflammatory cytokines IL-1α and TNF-α, and classical complement component C1q by activated retinal microglia, which induced reactive astrocytes to adopt a neurotoxic A1 phenotype in the retinae leading to RGC death after ONC in mice (112, 113). Interestingly, resting astrocytes promoted synaptogenesis in axotomized RGC cultures; however, RGCs failed to form synapses when co-cultured with A1 astrocytes. In addition, A1 astrocytes almost completely lose the phagocytic ability to phagocytose synaptosomes and myelin debris in cultures, suggesting potential ineffective synaptic pruning and myelin debris clearance which hampers the axonal remodeling and regrowth (112).

Sustained and unresolved inflammation in the retinae might also affect the permeability of blood-retinal barrier after ONC. The disruption or breakdown of blood-retinal barrier is a clinical feature of several ocular diseases including glaucoma, diabetic retinopathy, age-related macular degeneration, retinal vein occlusion, and uveitis (114, 115), which often lead to macular edema and retinal tissue damage due to increased vascular permeability (115). Accumulating evidence suggests that chronic neuroinflammation and elevated production of pro-inflammatory cytokines in the retinae contributed to the breakdown of blood-retinal barrier in diabetic retinopathy. Elevated levels of pro-inflammatory cytokines such as IL-1β, TNF-α and IL-6 were found in the retinae of patients with diabetic retinopathy (116–118), as well as in mouse or rat models of diabetic retinopathy (119–121). Intravitreal injections of pro-inflammatory cytokines markedly increased the permeability of blood-retinal barrier, and caused a massive influx of leukocytes into the retinae in both rats and rabbits (122–126). Interestingly, the blood-retinal barrier remained relatively intact up to 12 days after ONC (53). Nevertheless, it is yet to determine whether prolonged activation of retinal microglia and elevation of pro-inflammatory cytokines (IL-1β, IL-6 and TNF-α) (101, 102) contributed to the breakdown of blood-retinal barrier after ONC. In a mouse model of TON, expression levels of zonula occludens-1 and occluding (major tight junction proteins present in the blood-retinal barrier) were markedly reduced in the retinae, indicating the dysfunction of blood-retinal barrier. At the same time, elevated levels of pro-inflammatory cytokine TNF-α and IBA-1-positive activated microglia were detected in the retinae at 5 days post-injury (127).

Microglial activation and subsequent inflammation-mediated neurotoxicity are implicated as a target of therapeutic intervention to alleviate neuronal cell death and axonal injury. For instance, arginase 2 was elevated shortly (6 h) after ONC. Genetic ablation of arginase 2 markedly suppressed microglial activation in the injured retina and reduced the mRNA levels of IL-1β as well as its precursor (pro-IL-1β) protein expression. More importantly, arginase 2 ablation and the attenuation of microglial activation protected injured RGCs from apoptosis, and induced a considerable amount of axon regeneration at day 14 post-ONC (128). Oral administration of spermidine, an endogenous free radical scavenger that protected the cells from oxidative stress, markedly inhibited microglial activation and expression of chemokines such as CCL2 and CCL5 after ONC. The expression of iNOS was also suppressed in the activated microglia resulted in greater RGC survival and modest yet significant axon regeneration two weeks after ONC (129). Neutralizing antibodies specific for Sems3A abolished the Sema3A actions and switched the retinal microglial polarization from neurotoxic M1-like phenotype to neuroprotective M2-like phenotype at 3-7 days post-ONC, which promoted RGC survival (102). Interestingly, microglial depletion using PLX5622 exhibited a transient neuroprotective effect against RGC death and axon degeneration at day 5 after ONC (105), possibly due to the reduction of microglia-derived semaphorin 3A (Sema3A) and M1-activated microglia in the retinae (102). However, depletion of retinal microglia using PLX5622 did not further enhanced or reduced RGC survival at later stage (days 7-21) after ONC (94). A recent study showed that by inhibiting the activity of mammalian target of rapamycin (mTOR) using resveratrol (sirtuin-1 activator which inhibits mTOR activity) (130) or by genetic ablation of microglia-specific regulatory-associated protein of mTOR (Raptor) largely suppressed the colonization of activated microglia, which led to a reduced level of IL-1β in proximal nerve stump after ONC. RGC loss and axonal damage was reduced in mice with resveratrol treatment or Raptor deletion in microglia at day 5 post-ONC (105). In another study, intravitreal injection of neutralizing antibodies against microglia-specific secretory factors IL-1α, TNF-α and C1q inhibited the neurotoxic A1 astrocytes formation and the subsequent RGC death after ONC in mice (112, 113). These studies collectively suggest that by counteracting the detrimental role of microglia, instead of complete removal of resident microglia from injured retinae and optic nerve, could elicit a potent neuroprotective and pro-regenerative effect after ONC.

### Controlled Neuroinflammation Promotes Axon Regeneration After Optic Nerve Injuries

Lens injury primes adult RGCs into an actively growing state as a result of the activation of resident retinal microglia, astrocytes and Müller cells, and infiltration of peripheral myeloid cells into the retinae, leading to enhanced RGC survival and axon regeneration after ONC in both mice and rats (83, 131, 132). Intravitreal injection of zymosan, a cell wall suspension from yeast, elicits a massive infiltration of myeloid cells into the retina and recapitulates the lens injury-induced growth-promoting effects (83, 84, 99). Meanwhile, the infiltration of myeloid cells into the retinae also activated retinal astrocytes and Müller cells, leading to the production of Müller cell-derived growth factors and robust axon regeneration after intravitreal injections of zymosan and ONC. In stark contrast, intra-optic nerve zymosan injection failed to elicit infiltration of myeloid cells into the retinae and thus the activation of retinal astrocytes and Müller cells became very limited, resulting in poor axon regeneration after ONC (133). These results suggest that there may be crosstalk among resident retinal microglia, astrocytes, Müller cells, and infiltrated myeloid cells in the injured retinae for mediating inflammation-induced growth-promoting effects. However, current knowledge about the major factors contributing to the lens injury-induced and zymosan-induced growth-promoting effects remain limited. For instance, axotomized adult rat RGCs exhibited longer neurites when co-cultured with lens and vitreous body from zymosan-treated mice than vehicle-treated mice, since a large number of infiltrating macrophages were observed in the vitreous body after zymosan treatment. (132). Similarly, conditioned medium derived from zymosan-treated rat macrophage cultures promoted neurite outgrowth from axotomized adult rat RGCs (84). In a follow-up study, oncomodulin was identified as one of the major macrophage-derived growth factors for RGCs, which play a key role in the pro-regenerative processes of injured RGCs (84–86, 134). Intravitreal injections of recombinant oncomodulin with concurrent elevation of cyclic AMP (cAMP) enhanced binding of oncomodulin to RGCs (88) that recapitulated the zymosan-induced growth-promoting effects on axon regeneration (134). Nevertheless, Zymosan treatment alone did not induce sustained axon regeneration to grow over long distances to reinnervate visual target and thereby resulted in poor visual function recovery (89). Combinatorial treatments with zymosan and PTEN deletion that induced robust axon regeneration through activation of mTOR-mediated signaling pathway (61, 135), allowing axon to regenerate down the full length of the optic nerve, and to reinnervate the distal subcortical brain regions including the suprachiasmatic nucleus (SCN), lateral geniculate nucleus (LGN), olivary pretectal nucleus (OPN) and superior colliculus (SC) after ONC in mice (88, 89). The regenerated RGC connections partially rescued visually guided behaviors such as optomotor responses and depth perception (89). Mechanistically, it has been shown that β-glucan/dectin-1 signaling cascade was identified as a critical mediator of zymosan-induced axon regeneration (136). Dectin-1 is a phagocytic receptor expressed on resident microglia and infiltrating macrophages in the retinae (136), which acts as one of the pattern recognition receptors for zymosan (137). Genetic ablation of dectin-1 abolished the zymosan-induced axonal growth after ONC (136). Intravitreal injections of curdlan, a water-insoluble β-glucan which formed complex with dectin-1 (138), recruited retinal microglia and infiltrating myeloid cells that highly expressed dectin-1 to the retinae and induced robust axon regeneration two weeks after ONC. Interestingly, the curdlan-induced axonal growth requires dectin-1 expression in both resident retinal microglia and infiltrating myeloid cells. Genetic ablation of dectin-1 in either resident retinal microglia or infiltrating myeloid cells markedly attenuated the curdlan-induced axonal growth after ONC (136) (**Figure 1C**). These studies collectively point to the direction that the production of secreted factors might contribute to the lens-injured induced growth-promoting effects by activating resident retinal glial cells such as retinal microglia and inflammatory responses elicited occur in the presence of infiltrating macrophages after ONC (139) that requires further investigation.

Lens injury and intravitreal injections of zymosan trigger intraocular inflammatory responses in the retinae leading to unprecedented levels of axon regeneration following ONC; however, depletion of resident retinal microglia using PLX5622 did not attenuate the degree of axon growth, suggesting the lack of involvement of resident retinal microglia in the pro-regenerative responses associated with lens injury. Also, co-treatment of PLX5622 with liposomal clodronate can be used to deplete both resident retinal microglia and infiltrating macrophages, which only shows modest impairment of intraocular inflammation that are required for axon regeneration (94). One possible explanation is that the presence of other glial cells such as astrocytes produce growth factors contributing to the lens injury- and zymosan-induced pro-regenerative processes. In fact, there was a profound up-regulation of ciliary neurotrophic factor (CNTF) and LIF expression in the reactive astrocytes at the GCL of the injured retinae (58, 140). Deletion of either CNTF or LIF alone, or co-deletion of both, or deletion of STAT3 (the downstream effector of CNTF), abolished the growth-promoting effects of lens injury or zymosan treatment (58, 140, 141). It has been reported that CNTF activated STAT3 in the RGCs *via* the JAK/STAT signaling pathway, and the activation of JAK/STAT signaling pathway might mediate the neuroprotective effects of CNTF on RGCs directly (142, 143). Intravitreal CNTF administration induced the recruitment of activated retinal microglia and infiltrating macrophages to facilitate its growth-promoting effects on RGC survival and axon regeneration after ONC in adult rats. Depletion of infiltrating macrophages by liposomal clodronate completely abolished the growth-promoting effects of CNTF (144). Yet researchers have long been baffled by CNTF contradictory effects on RGCs. Recombinant CNTF promoted axon outgrowth from neonate RGCs in culture (145), but failed to exert similar effect on cultured mature RGCs (134). Several studies reported that recombinant CNTF promoted optic nerve regeneration (58, 140), however; others found very limited promoting effects unless suppressor of cytokine signaling 3 (SOCS3) was deleted in the RGCs (146, 147). Effective administration of CNTF to RGCs may be hampered by the exceedingly short half-life of CNTF (148). In fact, the use of viral-based vector to transfer CNTF into injured retinae has proven to be a promising method for long-term expression of CNTF results in a robust axon regeneration (149–152), possibly *via* up-regulation of C-C motif chemokine ligand 5 (CCL5) in macrophage and infiltrating neutrophil. Depletion of neutrophil or pharmaceutical blockade of CCL5 receptor CCR5, completely abolished the growth-promoting effects induced by CNTF overexpression (152). Another plausible explanation for the ineffectiveness of microglia/macrophage depletion to compromise lens injury-induced axon regeneration is that macrophage might not be the only immune cell producing oncomodulin. Previous studies showed that infiltrating macrophages and neutrophils in the aqueous and vitreous humor produced oncomodulin after lens injury or zymosan treatment (85, 86, 134). Blocking the binding of oncomodulin to its receptors or depletion of neutrophils decreased the number of axons regenerating into the distal optic nerve after zymosan treatment, but did not completely abolish the promoting effects (85, 86, 88). High expression of oncomodulin was detected in both GCL and IPL in the retina by immunohistology (134), quantitative RT-PCR, and Western blot analysis (85). Nevertheless, the oncomodulin-positive cells did not overlap with ED-1-positive microglia or infiltrating macrophages (134). It suggests that, other than infiltrating microglia/macrophages, there might be other cells that produce oncomodulin after lens injury or zymosan treatment. Recently, a conditional microglia/macrophage-specific CX3CR1-Cre mouse line, which expresses Cre recombinase under the control of a chemokine receptor CX3CR1 promoter, was established for studying microglia/macrophage (153). By crossing the CX3CR1-Cre transgenic mouse line with a oncomodulin-floxed mouse line (154), oncomodulin could be deleted specifically from most of the resident retinal microglia and infiltrating macrophages. Using the conditional gene knock-out approaches, one might conclude that if infiltrating myeloid cells were the major source of oncomodulin. It might help to address the controversies regarding the importance of oncomodulin and microglia/macrophage activation in promoting axon regeneration (155, 156).

Long-distance axon regeneration induced by combinatorial treatments of zymosan and PTEN deletion partially restored optomotor responses and depth perception; however, most of the mice developed severe cataracts months after ONC possibly due to chronic and unresolved intraocular inflammation (89). Mice with cataracts were unable to regain a proper pupillary light reflex (PLR) and to constrict the pupil in response to light stimulation (89), despite the regenerated axons have already reached the sub-cortical brain target OPN that is responsible for PLR (157, 158). Zymosan and curdlan treatment induced a massive influx of infiltrating macrophages and activation of resident microglia, which caused retinal folding and detachment in the treated retinae (136), a condition similar to experimental autoimmune uveitis (159). Of note, although zymosan treatment triggered intraocular inflammation that promoted RGC survival and robust axon regeneration (83), some secretory factors generated by the infiltrating macrophages exhibited strong growth-inhibitory effects on neurite outgrowth and survival in axotomized adult RGCs *in vitro* (84). Therefore, a more in-depth study is required to identify myeloid cell subtypes and its associated growth factors, which induce axon regeneration and nerve repair without causing any tissue damages.

### Microglial Activation and Its Involvement in Re-Myelination After Optic Nerve Injuries

Some approaches that promote long-distance axon regeneration also induce a considerable degree of remyelination and support functional restoration of some simple visual tasks after ONC (89, 160). However, remyelination does not necessarily occur immediately after the injured RGC axons regenerated the entire length of the optic nerve. For instance, combinational genetic manipulations by co-deletion of PTEN and SOCS3 and overexpression of CNTF, or overexpression of osteopontin, IGF-1 and CNTF enabled long-distance axon regeneration and formation of functional synapses with their distal brain targets. These regenerating axons provoked significant neural activity at the superior colliculus upon stimulation to the injured axons. However, the regenerated axons remained poorly myelinated leading to poor recovery of visual-associated behaviors such as optomotor acuity after ONC (150), suggesting remyelination is required for visual function restoration.

Optic nerve transection led to a massive loss of oligodendrocytes in adult mice and rats (50). For myelination to occur after CNS injuries, oligodendrocyte precursor cells (OPCs) need to undergo rapid proliferation, differentiate into mature oligodendrocytes and ensheath the regenerating axons for proper nerve conduction (161, 162). Accumulating evidence suggested that resident microglia were involved in the remyelination process after CNS injuries (163). In a mouse model of demyelination, M1 (TNF-α/iNOS-positive) microglia and macrophages were dominant at the early time point when OPCs were recruited and proliferated at the lesion site (164). These M1-polarized microglia and macrophages also expressed high levels of genes related to myelin debris clearance including C1q complement proteins and CX3CR1. Interestingly, these M1-polarized microglia and macrophages underwent necroptosis and most of them were eliminated before the onset of remyelination (165). At the time when OPCs initialized to differentiate into mature oligodendrocytes, these residual microglia and macrophages transformed into M2-like phenotype (165), and expressed distinct M2 markers arginase-1, CD206 and IGF-1 (164). Blocking the CD206 (M2) receptors suppressed M2 polarization in these microglia and macrophages, and inhibited oligodendrocyte differentiation. In contrast, promoting M2 polarization of these microglia and macrophages accelerated the remyelination process (164), suggesting that microglial polarization might be a key determinant for successful oligodendrocyte differentiation and remyelination.

A recent study showed that OPCs were rapidly proliferated at the crushed optic nerve in the first five days, and gradually declined to basal levels at the later time points after ONC. This process largely depends on activation of resident retinal microglia as depleting these activated microglia immediately after ONC largely inhibited the rapid proliferation of OPCs in the first two weeks after ONC. Unfortunately, these proliferating OPCs failed to differentiate into mature oligodendrocytes for remyelination even though robust axon regeneration occurred after growth stimulation by overexpression of osteopontin, IGF-1 and CNTF. One possible reason for this remyelination failure is that the resident retinal microglial remained activated even at later time points (i.e. 21 days) after ONC. These activated microglia adopted a M1-like phenotype and expressed a high level of iNOS. Delayed elimination of activated microglia in the injured optic nerve at 2 weeks post-ONC not only allowed the OPCs to proliferate, but also promote maturation of these OPCs to differentiate into mature oligodendrocytes for remyelination at 6 weeks post-ONC (101). Of note, promoting myelination is crucial to protect the regenerating axons from degeneration and sustain long-distance axon regeneration towards the distal brain targets for reinnervation. Most of the unmyelinated regenerating axons started to degenerate if they failed to innervate their targets in a short period of time (101). Therapeutic interventions that promoted M2 microglial polarization might be beneficial to facilitate robust remyelination after ONC (163).

## The Roles of Bipolar/Rod-Shaped Microglia in Traumatic Optic Neuropathy

The roles of ramified and amoeboid microglia have been extensively studied under normal and pathogenic conditions. However, little is known about the functional role of bipolar/rod-shaped microglia largely due to the lack of *in vitro* system to enrich bipolar/rod-shaped microglia (166, 167) and a highly reproducible *in vivo* model (168–170). Bipolar/rod-shaped microglia were first classified as an activated form of microglia by Nissl in 1899 (171) with slim cell bodies and indefinitely long processes. Bipolar/rod-shaped microglia were identified in postmortem cerebral cortices from patients with paralytic dementia, syphilis, typhus infections, and sleeping disorders (171, 172). The occurrence of these infectious diseases was dramatically reduced due to the rapid development of penicillin and other antibiotics, and thus reports on these bipolar/rod-shaped microglia became scarce until they were “rediscovered” in an experimental model of traumatic brain injury in adult rats (173). After a midline fluid percussion injury (mFPI), the resting ramified microglia quickly adopted a ‘rod-like’ morphology and colonized in primary somatosensory barrel field (S1BF) which formed trains of end-to-end alignment spanning perpendicular to the dura surface across the cortical layers at day 1 post-injury (173, 174). The formation of the trains of bipolar/rod-shaped microglia became more pronounced and with its peak at day 7 post-injury (173). Bipolar/rod-shaped microglia are morphologically distinct from other microglia subtypes, with an increase in cell length to width ratio and significantly fewer side processes (174). These bipolar/rod-shaped microglia formed alignment in close proximity to injured axons and expressed a high level of CD68 (a phagocytic marker) (173). The formation of the end-to-end alignment of bipolar/rod-shaped microglia was also found in other neuropathological disorders such as Alzheimer’s Disease, Parkinson’s Disease, Huntington’s Disease, viral encephalitis, lead encephalopathy, subacute sclerosing panencephalitis, and ischemic stroke. The possible functional roles of the bipolar/rod-shaped microglia in these disorders have been extensively discussed in recent reviews by us and others (168–170). The current Review only focuses on the role of bipolar/rod-shaped microglia in retinal disorders, especially on the pathological conditions in TOP.

Compelling evidence suggested that traumatic injuries to the optic nerve and retinal degeneration induced the formation of end-to-end alignments of bipolar/rod-shaped microglia in the retinae. In a rodent ocular hypertension (OHT) model of glaucoma, microglia quickly adopted bipolar/rod-shaped morphology with their processes aligned end-to-end in the NFL of OHT retinae, but not the contralateral eye (175). The bipolar/rod-shaped microglia in the OHT retinae exhibited high immunoreactivity of CD68 (phagocytic marker) and MHC-II (marker for antigen-presenting cells), but relatively low immunoreactivity of CD86 (M1 marker) and Ym1 (M2 marker) (175, 176). In contrast, amoeboid microglia were found in the OHT retinae with high immunoreactivity of both CD86 and Ym1 (176), whereas ramified microglia were restricted in the contralateral eye with low immunoreactivity of CD68 and MHC-II (175). The results suggest that bipolar/rod-shaped microglia are immunocompetent cells with distinct immunophenotypic profiles when compared with ramified (resting) and amoeboid (activated) microglia. After optic nerve transection in rats, microglia became activated and gradually adopted an amoeboid morphology as soon as at day 1 post-injury (38, 177). Amoeboid microglia actively engulf dying RGCs which have gradually lost the expression of Brn3a (RGC marker) (38). Interestingly, some of the activated microglia begin to transform into bipolar/rod-shaped microglia with elongated cell bodies and form end-to-end alignments in the GCL and NFL of retinae after ONC (**Figure 1B**). Trains of bipolar/rod-shaped microglia started to appear at day 3 post-injury, became more prominent at days 7 to 21 post-injury, and gradually disappeared at 6 weeks post-injury (38, 177). These bipolar/rod-shaped microglia were highly proliferative and aligned in close proximity to the βIII-tubulin-positive RGC axon fibres (177), in which they preferentially internalized the degenerating RGC axons, but not the RGC cell bodies (38, 177). Collectively, these studies suggest that bipolar/rod-shaped microglia are phagocytic cells that contribute to the clearance of degenerating RGC axons after optic nerve injuries.

Enriched ramified and amoeboid microglia are prepared by culturing primary microglia in a fibronectin-coated and laminin-coated surface, respectively (178). It allows researchers to examine the molecular and cellular responses of microglia with different morphologies to various cytokines, chemokines, chemoattractant, endotoxin, and so forth (168). However, the lack of a highly reproducible *in vitro* model system to enrich bipolar/rod-shaped microglia largely limited the studies on the functional characterization of this form of microglia in an isolated system. Recently, we have developed a cost-effective method to enrich bipolar/rod-shaped microglia *in vitro* with high reproducibility (166, 167). Primary microglia purified from postnatal mice were seeded onto a poly-D-lysine and laminin-coated surface with multiple scratches. Physical scratches removed laminin coating from the surface (167) and microglia colonized in the laminin-free scratched surface to form bipolar processes and end-to-end alignments in a direction parallel to the physical scratches (166, 167). Primary microglia colonized in the laminin-rich non-scratched surface displayed an amoeboid morphology that actively digested the surrounding laminin by up-regulation of laminin-cleaving proteins ADAM9 and CTSS (167). Compared with amoeboid-enriched primary microglia, bipolar/rod-shaped microglia were highly proliferative and exhibited decreased levels of both M1 (TNF-α, IL-1β, CD32 and CD86) and M2 (IL-10 and TGF-β) markers (166). Upon LPS challenge, bipolar/rod-shaped microglia could quickly transform into amoeboid morphology and elevate the expression of M1 markers (TNF-α and IL-1β) (166) and laminin-cleaving proteins ADAM9 and CTSS (167). Taken together, our *in vitro* model system successfully produces highly-enriched bipolar/rod-shaped microglia which are highly resemble the *in vivo* end-to-end alignment of bipolar/rod-shaped microglia in the brain after CNS injuries. Cultured bipolar/rod-shaped microglia exhibit key morphological and phenotypic characteristics (i.e. highly proliferative with distinct M1/M2 polarization) reminiscent of bipolar/rod-shaped microglia in S1BF after mFPI (173, 174) and injured retinae after axotomy (38, 177). In recent years, primary retinal microglia have been successfully isolated and purified from the postnatal (179, 180) and adult (181) mice. We believe that our *in vitro* system could be used to enrich bipolar/rod-shaped microglia from primary retinal microglia for gene expression profiling and cellular property studies.

## Future Directions: The Distinct Roles of Resident Retinal Microglia on RGC Survival and Axon Regeneration

Traumatic injuries trigger activation of resident microglia, as well as a massive influx of blood-borne macrophages (38, 39). It remains a technical challenge to distinguish between resident microglia and infiltrating myeloid cells from the pool of activated microglia/macrophage residing in the injured retinae and optic nerves. Both resident retinal microglia and infiltrating myeloid cells expressed high levels of CD11b, CD45, F4/80, and IBA-1 (20, 182). One might manage to separate CD11b-positive resident microglia expressing a relatively low level of CD45 (i.e. CD11b^+^/CD45^low^) from those CD11b-positive infiltrating macrophages expressing a relatively high level of CD45 (i.e. CD11b^+^/CD45^high^) by fluorescence-activated cell sorting analysis (183). However, the expression of CD45 gradually increased in the resident microglia over the course of neuroinflammation, making it difficult to separate the resident microglia from infiltrating macrophages at the advanced stages of neuroinflammation (184). Despite the fact that both resident microglia and blood-borne macrophages share similar biological functions such as antigen presentation, phagocytosis of cellular debris, and production of inflammatory cytokines and chemokines (185). A recent study highlighted that there was a substantial difference between resident microglia and blood-borne macrophages in gene expression profiles. It implies that these two populations of cells have clear functional differences under normal and pathogenic conditions (186). Therefore, a microglia-specific marker is required to distinguish resident microglia from infiltrating myeloid cells and thus researchers can study the unique functions and cellular responses of resident microglia after CNS injuries.

A recent study identified TMEM119 as a microglia-specific marker that specifically labeled resident retinal microglia with no immunoreactivity in infiltrating myeloid cells. TMEM119 was exclusively expressed in the resident microglia in the brain parenchyma, but not expressed in blood-borne macrophages that abundantly residing in the liver and spleen (187). In a subsequent experiment, ONC was performed in CCR2-RFP mice (187) with which the red fluorescent protein (RFP) was expressed in infiltrating myeloid cells only (188). After ONC, two distinct populations of IBA-1-positive cells migrated to colonize the crushed site. A population of IBA-1-positive cells displayed distinctive TMEM119 immunoreactivity, but no RFP expression, were characterized as resident retinal microglia. Those IBA-1- and RFP-positive cells representing the population of infiltrating myeloid cells, which showed no TMEM119 immunoreactivity. It therefore suggests that TMEM119 is a microglia-specific marker labeling both resting and activated microglia (187).

In another study, P2RY12 was characterized as a resident microglia marker (186). The expression of P2RY12 was dramatically reduced during the progression of neurodegenerative diseases such as Alzheimer’s disease and multiple sclerosis (189–192), suggesting that the expression level of P2RY12 might be used as an indicator of resident microglia and activated microglia (i.e. TMEM119-positive and P2RY12-negative) after CNS injuries. A recently developed TMEM119-tdTomato and TMEM119-eGFP reporter mice enabled the isolation and purification of resident retinal microglia for transcriptomic studies (193, 194), which might facilitate the identification of potential growth mediators associated with activated microglia after intraocular inflammation. In addition, TMEM119-CreERT2 transgenic mice expressed Cre recombinase only in the resident microglia (193). By crossing the TMEM119-CreERT2 transgenic mice with transgenic mice expressing Cre-inducible diphtheria toxin receptor (DTR) (195), microglial depletion could be achieved after intraperitoneal injections of tamoxifen (to induce the expression of Cre recombinase in resident microglia and the expression of DTR) followed by a low dose of diphtheria toxin to deplete resident microglia. This transgenic mouse model allows the study of RGC survival and axon regeneration after ONC in the absence of resident retinal microglia without the need for a continued supply of PLX3397- or PLX5622-containing chow diet.

Current available strategies for analyzing gene expression perturbation from bulk cell population (e.g., CD11b^+^/CD45^low^ resident microglia and CD11b^+^/CD45^high^ infiltrating macrophages) are not sufficient to resolve cell-to-cell heterogeneity in complex immune responses (e.g., ONC and intraocular inflammation). Intraocular inflammation triggers the production of growth-promoting factors that accelerated axon regeneration after ONC (84, 85, 134), and growth-inhibitory factors that induce axon degeneration, secondary tissue damages and apoptotic cell death (84, 136). In the injured retinae, it is believed that a specialized subset of activated microglia and macrophages contribute to the production of neuroprotective agents, while the other subsets contribute to the production of neurotoxic factors. Transcriptome deconvolution allows the evaluation of relative changes in cell-type proportions after ONC based on transcriptomic data generated from the bulk microglial population, subject to the availability of readily available and well-characterized marker genes from each microglial subset (196, 197). In fact, the availability of single-cell genomic technologies such as single-cell RNA-sequencing enables a more comprehensive and unbiased molecular characterization of diverse microglial subsets under normal and pathogenic conditions. It also allows the identification of novel markers representing microglial subpopulations with different activation statuses (198). Using single-cell RNA-sequencing, a recent study successfully identified a novel microglial subtype, known as disease-associated microglia (DAM), which contributed to the progression of Alzheimer’s Disease. Upon the disease progression, resident microglia gradually lost their homeostatic identity with down-regulation of *P2ry12*, *P2ry13* and *Cx3cr1*, and increased expression of DAM marker genes (*Apoe*, *Lpl*, *Cd9*, *Cst7* and *Trem2*) (189). With the availability of several microglia-specific reporter mouse lines (193, 194), one can purify the resident retinal microglia from injured retinae and optic nerves, and to further identify microglial subtypes that contribute to the neuroprotective and growth-promoting effects after ONC. This strategy provides an important foundation for future development of novel therapeutic interventions by switching the resident retinal microglia’s phenotype to foster neuroprotection.

## Author Contributions

NPBA and CHEM discussed and developed the idea of the review. NA performed the literature review and wrote the first draft of the manuscript under the supervision of CHEM. CHEM revised the manuscript for final submission. All the authors have read the final version of the manuscript and approved for submission.

## Funding

This work was supported in part by a General Research Fund (GRF) from The Research Grant Council of the Government of the Hong Kong Special Administrative Region (CityU 11100519, and CityU 11100318); the Health and Medical Research Fund (HMRF), Food and Health Bureau, Hong Kong Special Administrative Region Government (07181356); and the National Natural Science Foundation of China (NSFC) (81971149) awarded to CHEM.

## Conflict of Interest

The authors declare that the research was conducted in the absence of any commercial or financial relationships that could be construed as a potential conflict of interest.

## Publisher’s Note

All claims expressed in this article are solely those of the authors and do not necessarily represent those of their affiliated organizations, or those of the publisher, the editors and the reviewers. Any product that may be evaluated in this article, or claim that may be made by its manufacturer, is not guaranteed or endorsed by the publisher.
